# Supplementary material for: Carbon footprint of the construction sector is projected to double by 2050 globally
Source: Commun Earth Environ. 2025 Oct 27;6(1):831. doi: 10.1038/s43247-025-02840-x (PMC12559003; doi:10.1038/s43247-025-02840-x)
Supplement: Supplementary file 1 — Supplementary Information [file 43247_2025_2840_MOESM1_ESM.pdf]

Supplementary Information
for

Carbon footprint of the construction sector is projected to double by 2050 globally

This file includes:

Supplementary Notes
Supplementary Methods
Supplementary Figures
References

Other Supplementary Material for this manuscript includes:

Data S1 includes tables on country, sector, and other input-output data details.
Data S2 contains tables on numerical outputs mentioned in the main text and supplementary information for this study.
Data S3 includes Tables on socio-economic datasets used in this study, regression results, and statistical tests.

Supplementary Note

Supplementary Note 1. Definition, scope, and system boundary of the construction sector

Here we define the construction carbon footprint as the carbon cost of constructing the built-environment. This includes the construction of all built-environment artifacts, including buildings (residential and non-residential), transport infrastructure (railroads, highways, subways, pavements, harbors, airports, tunnels, etc.), and technical infrastructure (communication infrastructure, underground pipes for waste disposal, telecommunication, energy generation systems, etc.). The accounted carbon footprints include direct (on-site) and indirect (supply chain) emissions associated with construction, including all materials (steel, cement, clinker, bricks and clay, biobased, glass, other metals, and all other miscellaneous materials), and all process involved in the supply chains (transport, services, machinery, capital formation, and onsite emissions). The construction carbon footprint does not include the emissions occurring after the completion of the construction stage, including the emissions during the operation of buildings, emissions generated from the energy infrastructures, etc.

Comprehensive usually comes with the cost of sacrificing details. Here, the details of sub-divisions of specific infrastructure types are being sacrificed, i.e., we are not able to distinguish how much emission originates from the construction of buildings/railroads/highways/subways/pavements/airports/power infrastructure, etc. Identifying these infrastructure type details on a global scale would be difficult, as this would mean an exponentially increasing number of included supply chains. We compensate for this by including full supply chain processes related to the construction industry as a whole, and providing full global coverage at a national resolution, and long timeseries span. This trade-off stems from our methodological approach with the goal of seeking a balance between comprehensiveness and details, by combining top-down methodology and using detailed input-output table with extensive supply chain information. Nevertheless, providing an estimate of the carbon cost of constructing the built-environment as a whole with supply chain and country-specific details provide useful information, at the same time acknowledging that further breaking down the infrastructure types would be a very interesting area of study for future research.

Supplementary Note 2. State-of-the-art

We here provide summary of the state-of-the-art based on a meta-review on built environment/buildings/infrastructure/construction.

First, studies typically have limited coverage in terms of geography, supply chain, and end-use categories. Specifically, global-scale studies are extremely rare, with most studies focusing on national, regional, or building-level scales[i]. In terms of supply chain coverage, many studies only examine a narrow range of materials, such as steel and cement, while neglecting non-material footprints[2]. Similarly, end-use category coverage is often restricted, with many studies focusing only on specific building/infrastructure types[3,4] or omitting infrastructure altogether[5]. Additionally, the system boundaries for end-use categories and the included supply chains in studies often vary significantly or are not clearly defined[1,6].

Second, dynamic studies analyzing changes over time, both retrospective and prospective, are scarce[7]. Retrospective dynamics refer to the analysis of historical time series of footprints, and prospective dynamics refer to projections of future trends. The scarcity of studies on the dynamic evolution of the construction industry significantly limits our understanding of its future trajectory. In the few studies that incorporate such dynamic time-series analyses, there is often an assumption of constant technology levels over time, or applying the same technological assumptions to both developed and developing regions[ii].

Third, the methodologies employed in existing studies differ significantly depending on data availability and the specific objectives of the research[iii]. These approaches are frequently inconsistent, making it challenging to compare and synthesize findings.

To summarize, the majority of studies in this field are micro-level studies, focusing on single-building level, regional, or national impacts. Global-level assessment of the construction carbon footprint is now greatly lacking. Much less is known about the

---

[i] For example, Onat (2020) reviewed 1833 documents relating to construction carbon footprints, of which 94% (1414 papers) of these papers are micro-level analysis[1]. Of the rest, another 93% of these are city level or national level, leaving only 6 papers on global analysis and one study on multinational analysis. Of these global level studies, they focus on only single year, with no information on sub-sectoral constitutions of what actually make up the carbon footprints, historical trends, or future projections, or multi-level footprint.

[ii] For example see Ref[8].

[iii] For example, systematic review by Mathuret al. (2021) show that most footprint assessment research on construction focus on a specific feature of the construction industry or single case studies in a particular region[6]. Even within these reviewed studies (105 papers), they differ in methodologies, units, system boundaries, and techniques of reporting. The review concluded that it is unrealistic to compare results from different studies, let alone synthesize results from different studies. The study also points out that although it is clear that the construction industry take up a remarkable percentage of the footprints, details or the holistic picture of this sector remain elusive.

composition of the footprints, cross regional/country comparisons, the historical trend, or the future trajectory of this sector. Comparison of results across nations and supply chains is difficult due to the use of different datasets, accounting years, and system boundary definitions.

Supplementary Note 3. Comparison with studies

Due to these, the studies available for comparison are scarce. We identified three global studies for comparison.

Müller (2013)[8] estimated that infrastructure would contribute 350 Gt of CO_2 emissions from material production between 2000 and 2050. In comparison, our estimate is higher at 440 Gt for the period from 2023 to 2050. This difference can be attributed to several factors: (1) Muller's analysis includes only three materials used in infrastructure construction—steel, cement, and aluminum—whereas our estimate incorporates all materials used in construction and accounts for supply chain footprints such as transport, services, and capital formation. (2) Muller's estimate is based on a single year accounting data (year 2008), whereas our approach reflects evolving trends over a long time series (1995-2022). (3) Lastly, Muller et al. applied the same intensity factors for both developing and developed regions, whereas we consider regional differences in technological production, scale of economy, and socio-economic factors, among others.

Huang et al. (2018) [9] accounts for emissions from renewable and non-renewable energy in the construction sector (gasoline, diesel, other petroleum, hard coal, nature gas, etc.), and estimates the carbon emissions amount to 5.7GtCO_2 as of 2009, while our estimates are 8.3GtCO_2 (for the year 2009, and 4.4GtCO_2-12.2GtCO_2 from 1995-2022). Our estimates are comparatively higher because Huang et al. do not consider the carbon emissions embodied in intermediate supply chains or capital investment in the construction process[iv]. These are essential elements for the construction industry as it is heavily material-reliant, spans long supply chains, needs various processing stages of intermediate inputs, and is heavily investment-reliant (see Text S2). Furthermore, the focus of Huang et al.'s paper is limited to analyzing the 26 renewable/non-renewable energy types used in the construction sector (gasoline, diesel, other petroleum, hard coal, natural gas, etc.) instead of the carbon footprint in the construction sector supply chain.

Ma et al. (2024)[5] provides a perspective piece for challenges and opportunities in the global net-zero building sector. Ma et al. (2024) show that 37% of global carbon emissions come from the building sector as of 2022. Our study shows that construction carbon footprint accounts for 33% of global carbon emissions as of 2022. While these results are similar, they

[iv] For details, please refer to Refs[10–13].

are not comparable in the strictest sense, given that Ma et al. account for only buildings but do not account for infrastructure footprints (while our study does) and include both operational and embodied footprints (while our study does not account for operational footprint). The system boundary of these two studies is therefore intersected but does not overlap with each other. These omissions and inclusions of accounted factors could potentially cancel out each other, leading to similar results. However, we note that Ma et al.'s study is a perspective piece with no accounting methodology provided.

Since our study also provided detailed supply chain data, we also make comparisons with studies that conduct footprint calculations for specific construction materials. Specifically, we pinpoint comparison with the material cement, and not for other materials. This is because (a) accounting studies for cement carbon footprint are already extensive and have generally reached a consensus in terms of accounting values, and (b) almost all cement is used in the construction of the built-environment (buildings, infrastructures, etc.), while for other materials, they are not only used in construction but are also used in a wide range of industrial process (steel in automobile, glass in light manufacturing, clay in furniture and kitchenware, etc.). Thus, carbon footprint in cement could be used as a proxy for comparison of carbon footprint accounting in this study.

Refs[14–16] show that cement accounts for 2.8Gtons/y, or ~8% of global CO_2 emissions as of 2020. Our estimates show that cement accounts for around 2.9Gtons/y of CO_2 footprints as of 2020 (Data S2). This shows high consistency with estimated carbon footprints embodied in cement.

Supplementary Methods

Supplementary Methods 1. Further Explanations on Sector Classification

We categorize industries in the Input-Output table that are relevant to the construction industry and its environmental impacts through structured classification of industries based on their material usage and role in the construction life cycle. Specifically, the categorization aims to delineate the environmental pressures stemming from primary materials used in construction, such as steel, cement, glass, and bio-based materials, as well as the supporting industries that contribute indirectly through services, investment, transportation, and machinery. The industry classification is as follows: steel, cement, clinker, bricks and clay, biobased, glass, other metals, transport, services, machinery, investment, and onsite (see Table S1-2 in Data S1 for the full list of industries and corresponding sector numerations).

This study categorizes sectors relevant to the construction industry and its environmental impacts through a structured classification of industries based on their material usage and role in the construction lifecycle. Specifically, the categorization aims to delineate the environmental pressures stemming from primary materials used in construction, such as steel, cement, glass, and bio-based materials, as well as the supporting industries that contribute indirectly through services, investment, transportation, and machinery. The detailed sector classification is as follows: 1) Steel: Includes the mining of iron ores (Sector 25), the manufacturing of basic iron and steel (Sector 72), and the re-processing of secondary steel into new steel (Sector 73). 2) Cement: Comprises the manufacture of cement, lime, and plaster (sector 69). 3) Clinker: Includes the re-processing of ash into clinker (sector 70) and the manufacture of other non-metallic mineral products (sector 71). 4) Biobased: Includes wood and plant-based materials, plant-based fibers (sector 7), cultivation of crops n.e.c. (not elsewhere classified) (sector 8), wool and silk-worm cocoons (sector 15), forestry, logging, and related activities (sector 18), and the manufacture of wood and related products (sectors 50 and 54). 5) Glass: Covers the manufacture of glass and glass products (sector 65) and the re-processing of secondary glass into new glass (sector 66). 6) Other Metals: Includes the mining and processing of a variety of non-ferrous metals, such as copper (sector 26), aluminum (sector 28), lead, zinc, and tin (sector 30), and related re-processing activities (sectors 74–85). 7) Transport: Encompasses transport via railways, pipelines, sea, air, and auxiliary transport services (sectors 120–126). The model calculates the different intensities associated with the transport of various materials in different regions with the algorithm. The derived intensity thus reflects the transport of different materials in different sectors as well as countries. 8) Bricks and Clay: Comprises the manufacture of ceramic goods (sector 67) and the manufacture of bricks, tiles, and construction products in baked clay (sector 68). 9) Services: Includes a wide

range of services related to construction, such as real estate, finance, insurance, and other auxiliary services (sectors 115–119, 127–138, and 159–163). 10) Light equipment: Covers the manufacture of lightweight equipment and manufacturing devices, such as chain saws, cutters, nail guns, etc. (sectors 86–93). 11) Capital asset: Covers capital investment in heavy machinery and infrastructure, such as investment in the development of new capital stock, heavy machinery such as cranes, loaders, concrete mixers, etc. (sector primary inputs). 12) Onsite construction: Includes emissions that occurred during onsite construction activities (sectors 113 and 114).

Supplementary Methods 2. EXIOBASE environmental satellite data (direct emissions/resource use)

The input-output tables used in this study are from the latest version of the EXIOBASE v3.8. This input-output table classifies the world economy into 44 countries and 5 RoW (rest of world) regions (see Table S3-4). This input-output table extends from 1995 to 2022. This input-output table classifies the world economy into 163 industries (see Table S1-2 in Data S1), which is one of the most detailed input-output tables available. EXIOBASE 3.8.2[17] encompasses direct resources and emission data from distinct industries within each country, which serves as the environmental module input for the MRIO model. Satellite accounts for the MRIO tables are based on data from the Food and Agricultural Organization (FAO), the International Energy Agency (IEA), the United Nations System of Environmental-Economic Accounting for Water (UN-SEEA Water)[18], International Recommendations for Water Statistics, amongst other sources (see Text SX for further details).

EXIOBASE 3.8.2[17] encompasses direct resources and emission data from distinct sectors within each country, which serves as the input for the MRIO model. The EXIOBASE dataset includes direct (onsite) greenhouse gas emissions, particulate matter, hazardous emissions, land use, energy use, water use, and water pollutants across various economic sectors. Prices are kept at current constant price in EXIOBASE data to isolate pseudo trend caused by inflation[17]. In this section, we summarize the methodological approach employed by EXIOBASE.

Emissions are obtained via a bottom-up approach, incorporating activity data and emission factors[19]. Emission coefficients are then determined at the level of detail provided by the Energy Supply-Use Tables (ESUTs) and Physical Supply-Use Tables (PSUTs). The calculated emissions are then juxtaposed with ESUT and PSUT data using emission coefficients, ensuring consistency with the accounting rules of the System of Environmental-Economic Accounting (SEEA) and with the energy and physical data in EXIOBASE. Fuel combustion and non-combustion activities are detailed, following the emission reporting requirements set out by international conventions. Emissions from combustion processes are calculated by combining the emission coefficients with energy use data that align with the system boundaries of the SEEA. Procedures for calculating emissions related to these activities are guided by the IPCC's guidelines[20]. The emissions from waste treatment activities are determined based on the method described in ref [21].

Supplementary Methods 3. Further Explanations on Panel Projections

Here, we further explain the panel regression models we used to forecast construction footprints into 2050. The projection is done in several steps. First, we utilize OLS and fixed effect regression models to check for relevant factors in projecting future construction sectors. We do this by running a series of fixed effect models to check GDP, population, urban population, and industry value added, among other factors influencing the historical construction sector footprint. Since regressing variables with similar trends will lead to multicollinearity issues, we check for the best variable that predicts footprint growth based on series of models that regress various socio-economic factors and historical construction footprints. Through these models, we show that population best explains historical construction footprint based on significance levels and R squared values[V]. Regressing GDP, population, and urban population at the same time will result in multicollinearity and large uncertainty with wide confidence intervals, as they all have similar trends (see Data S3).

Next, a series of tests is run to ensure the model runs without multicollinearity, test for heteroscedasticity, and so on. We do this by checking the variance inflation factor (VIF) to detect multicollinearity, removing highly correlated variables, investigating sources of variability or noise in the data, and testing for heteroscedasticity through ARCH and GARCH tests (for details, see Supplementary Methods 4 and Data S3).

Subsequently, we utilized regression models to forecast construction footprints into 2050. We sourced the data from a Shared Socioeconomic Pathways database and World Bank, which contained historical and projected data on population. Population projections were aligned with the Shared Socioeconomic Pathways (SSPs 1-5) to represent different future socioeconomic scenarios. Missing values in the population datasets were handled via linear interpolation, ensuring a continuous time series for accurate model fitting. The general linear regression model used for analysis is as follows:

$$\text{Footprint}_{i,t} = \beta_1 \text{ Population }_{i,t} + \epsilon_{i,t} \quad (1)$$

$$\text{Footprint}_{i,t} = \beta_1 \text{Urban}_{i,t} + \epsilon_{i,t} \quad (2)$$

(3)

[V] R squared values are commonly used in econometrics modelling that indicates how much of the variation of a dependent variable is explained by an independent variable.

$$\text{Footprint}_{i,t} = \beta_1 \text{GDP}_{i,t} + \epsilon_{i,t}$$

(4)

$$\text{Footprint}_{i,t} = \beta_1 \text{GDP}_{i,t} + \beta_2 \text{ Industry }_{i,t} + \epsilon_{i,t}$$

(5)

$$\text{Footprint}_{i,t} = \beta_1 \text{GDP}_{i,t} + \epsilon_{i,t}$$

(6)

$$\text{Footprint}_{i,t} = \beta_1 \text{GDP}_{i,t} + \beta_2 \text{ Population }_{i,t} + \epsilon_{i,t}$$

(7)

$$\text{Footprint}_{i,t} = \beta_1 \text{GDP}_{i,t} + \beta_2 \text{ Industry }_{i,t} + \beta_3 \text{ Urban }_{i,t} + \beta_4 \text{ Population }_{i,t} + \beta_5 \text{GDP}^2_{i,t} + \epsilon_{i,t}$$

where:

i represents the country (or panel unit), t represents the year, $\epsilon_{i,t}$ is the error term. Footprint t_t is the environmental footprint (carbon, energy, or water) at time t, population $_t$ is the population value at time t (both historical and projected values under SSPs), β_n are the regression coefficients, and ϵ_t is the error term.

Separate regression models were built using historical population data and future projections for SSP1 to SSP5, allowing us to capture potential environmental impacts under various future demographic scenarios. Using the trained regression models, we predicted future environmental footprints based on population projections under the SSP1-5 scenarios. Additionally, 95% prediction intervals were calculated to estimate the range of uncertainty associated with these forecasts. To assess the performance of the models, the predicted values were compared to actual historical data points. The accuracy of the regression models was evaluated based on how closely the predicted values matched the actual data.

We note that regional projections carry greater uncertainty than global projections and are therefore treated separately. For instance, China's population is already declining, yet its GDP is projected to continue rising. Whether China's future construction footprint grows, declines, or stabilizes will depend on whether the sector is driven more by economic stimulus or demographic demand, reflecting the housing policy pathway chosen. Here, we assume that China's future construction will be more impacted by population growth, but it is highly plausible that it will remain strong due to economic stimulus. Our estimation for China is therefore a conservative estimate. Regional projections are thus kept independent to account

and isolate the broader range of factors and model sensitivities that shape outcomes at this smaller scale. Regardless, our main finding of a doubling in the future footprint is based on global projections, which are highly robust across models and exogenous control variables, which stems from the near-linear and consistent historical growth of the construction industry, as well as long-established and coordinated trends in population and economic growth.

Supplementary Methods 4. Further explanations on statistical tests prior to regression.

Before conducting regression analysis, we perform several statistical tests to ensure that the data meets essential assumptions: stationarity, homoscedasticity, and the absence of autocorrelation. To check for stationarity, we utilize the KPSS (Kwiatkowski-Phillips-Schmidt-Shin), Phillips-Perron (PP), and Augmented Dickey-Fuller (ADF) tests. For autocorrelation, we apply the Pearson Correlation Test, Ljung-Box Test, and Durbin-Watson Test. Additionally, we assess heteroscedasticity using the ARCH and GARCH tests. These procedures confirm that our regression assumptions are satisfied, which leads to more reliable results. By ensuring stationarity, eliminating autocorrelation, validating homoscedasticity, and confirming linear relationships, our regression analysis adheres to econometric standards. Further details and test results can be found in Data S3.

Stationarity refers to the property of a time series where its statistical attributes—such as mean, variance, and covariance—remain constant over time. To confirm that our data meets this requirement, we employ three tests: the KPSS test, the Phillips-Perron (PP) test, and the Augmented Dickey-Fuller (ADF) test. The KPSS (Kwiatkowski-Phillips-Schmidt-Shin) test is used to assess the null hypothesis that the time series is stationary around a deterministic trend against the alternative that it contains a unit root. Essentially, it helps differentiate between trend-stationary series and those with a unit root. Combining this test with the unit root hypothesis can more accurately classify the time series as stationary, non-stationary, or inconclusive. In addition to the KPSS, we employ the PP and ADF tests. Both the Phillips-Perron and Augmented Dickey-Fuller tests are designed to test the null hypothesis of a unit root, where a rejection of the null indicates that the series is stationary. These tests add further robustness to our analysis by confirming that no unit roots are present, thereby reinforcing the stationarity of the data. All three tests—KPSS, PP, and ADF—return positive results, confirming that the time series data is stationary and, therefore, suitable for further econometric modeling (refer to Data S3 for detailed test outcomes).

Autocorrelation refers to the correlation of a signal with a delayed copy of itself as a function of delay. In time series data, autocorrelation can lead to misleading results in regression analysis, violating the assumption of independence among residuals. To ensure no autocorrelation is present, we apply both the Ljung-Box test and the Durbin-Watson test. The Ljung-Box test checks for autocorrelation in residuals up to a specified number of lags. A non-significant result indicates no significant autocorrelation exists at the tested lag levels. The Durbin-Watson test provides a statistic between 0 and 4, where a value close to 2 suggests no autocorrelation in the residuals. Both tests confirm that our regression models do not suffer from autocorrelation, further validating the reliability of the results.

Heteroscedasticity, or non-constant variance of residuals, violates another key assumption

of regression analysis. To address this, we apply the ARCH and GARCH models, which are designed to detect and model time-varying variance. A significant result would suggest that the residuals' variance changes over time, indicating heteroscedasticity. However, the results of these tests show no signs of heteroscedasticity across the models, confirming that the variance of the residuals remains constant.

Overall, the positive results of the stationarity, autocorrelation, and heteroscedasticity tests demonstrate that the assumptions for valid regression analysis are met. This provides a strong foundation for interpreting the regression results and drawing reliable conclusions. The tests ensure that the relationships identified between the variables are robust and free from statistical anomalies that could otherwise compromise the findings. (See Data S3 for detailed test results.)

Supplementary Methods 5. Further explanation of SSP framework and projection model

SSP1 assumes a world with sustainable development where population growth stabilizes, leading to reduced demand for new construction compared to other scenarios. Conversely, SSP2 depicts a middle-of-the-road scenario with moderate population growth and corresponding construction sector expansion. SSP3 projects a fragmented world with slower economic growth and high regional disparities, resulting in uneven demand for construction across regions. SSP4 envisions a world with deep inequalities, where a highly industrialized elite drives rapid urbanization and construction in affluent regions, while less developed areas experience stagnation and minimal infrastructure development. SSP5 anticipates rapid economic growth driven by fossil fuels, with a significant surge in construction activity due to rapid urbanization and population expansion. These variations in socio-economic pathways will shape the future trajectory of the construction sector under each SSP scenario.

The Shared Socioeconomic Pathways (SSPs) data used in this study were obtained from the SSP database, designed through collaboration between the Integrated Assessment Modeling (IAM) and the Impacts, Adaptation, and Vulnerability (IAV) communities. Population and urbanization projections were developed by the International Institute for Applied Systems Analysis (IIASA) and the National Center for Atmospheric Research (NCAR). For economic development (GDP), three different interpretations were provided by teams from IIASA, the Organization for Economic Co-operation and Development (OECD), and the Potsdam Institute for Climate Impact Research (PIK).

Supplementary Methods 6. Further explanation on carbon budget modeling

A carbon budget represents the maximum amount of cumulative CO_2 emissions that can be released while still having a certain likelihood of limiting global warming to these temperature targets. The approach for carbon budget modeling for per-annum trajectory adopted in this paper follows ref[22–24] but we use the most up-to-date data, based on a starting year 2023.

The carbon budget for limiting global warming to 1.5°C ranges from 500 billion tonnes of CO_2 with a 17% probability, to 300 billion tonnes for a 33% probability, 250 billion tonnes for a 50% probability, 150 billion tonnes for a 67% probability, and 100 billion tonnes for an 83% probability. For the 2°C scenario, the carbon budget is more lenient, starting at 2000 billion tonnes for a 17% probability, 1450 billion tonnes for a 33% probability, 1150 billion tonnes for a 50% probability, 950 billion tonnes for a 67% probability, and 800 billion tonnes for an 83% probability. For data and figures for these modeling values see Fig. S1-2.

Supplementary Figures

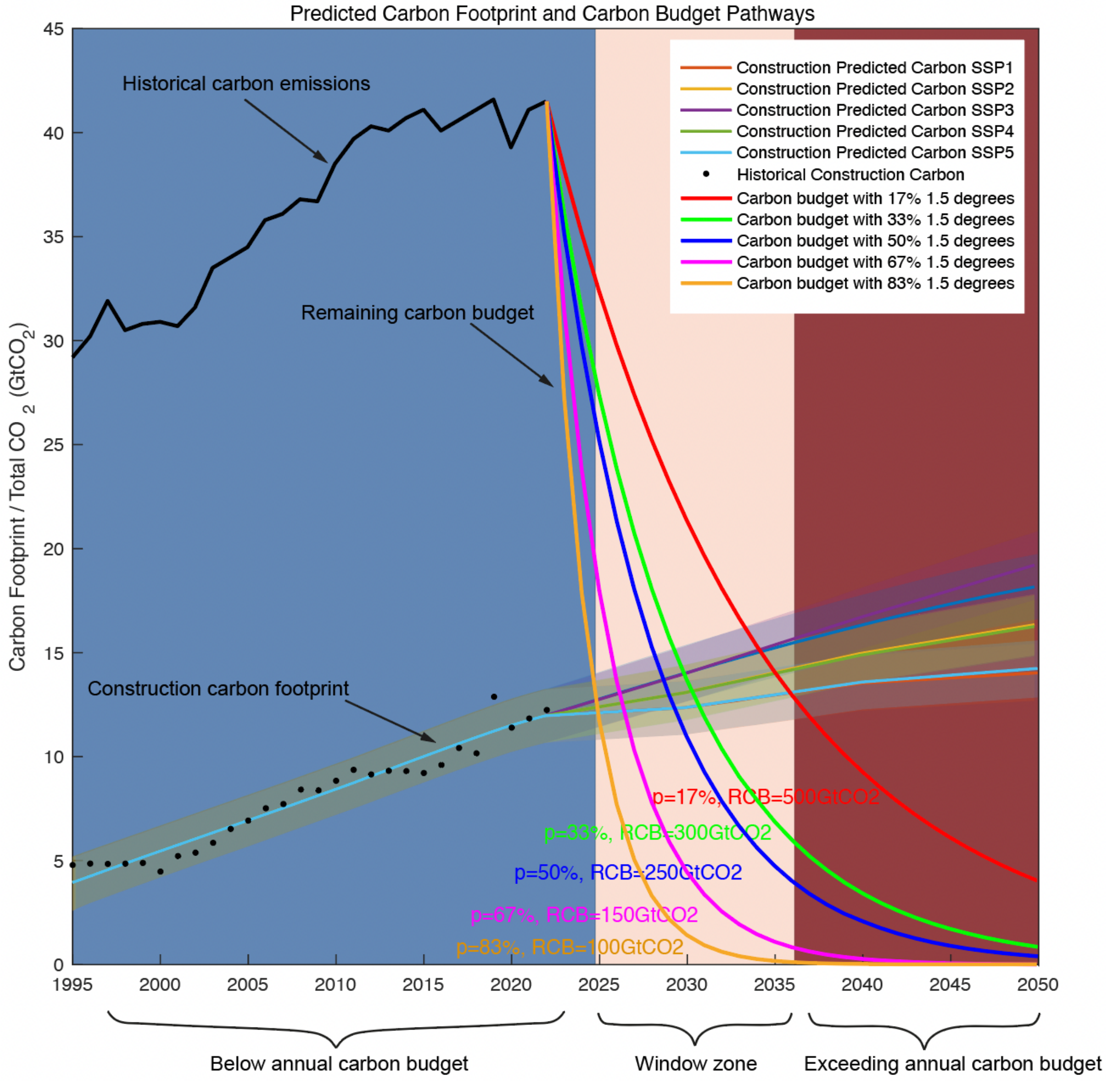

Fig. S1 Construction footprint projections and emission paths for reaching the 1.5 degrees Paris Agreement. Future annual carbon budget is modeled to achieve the 1.5 degrees Paris Agreement under 17%, 33%, 50%, 67%, and 83% possibilities. Future predictions of construction footprint are projected under different scenarios of Shared Socioeconomic Pathways (SSPs). Two pathways intersect in the 2025-2040 window zone. Beyond this, construction sector alone will use up all per-annum carbon budgets for 1.5 degrees latest by 2040.

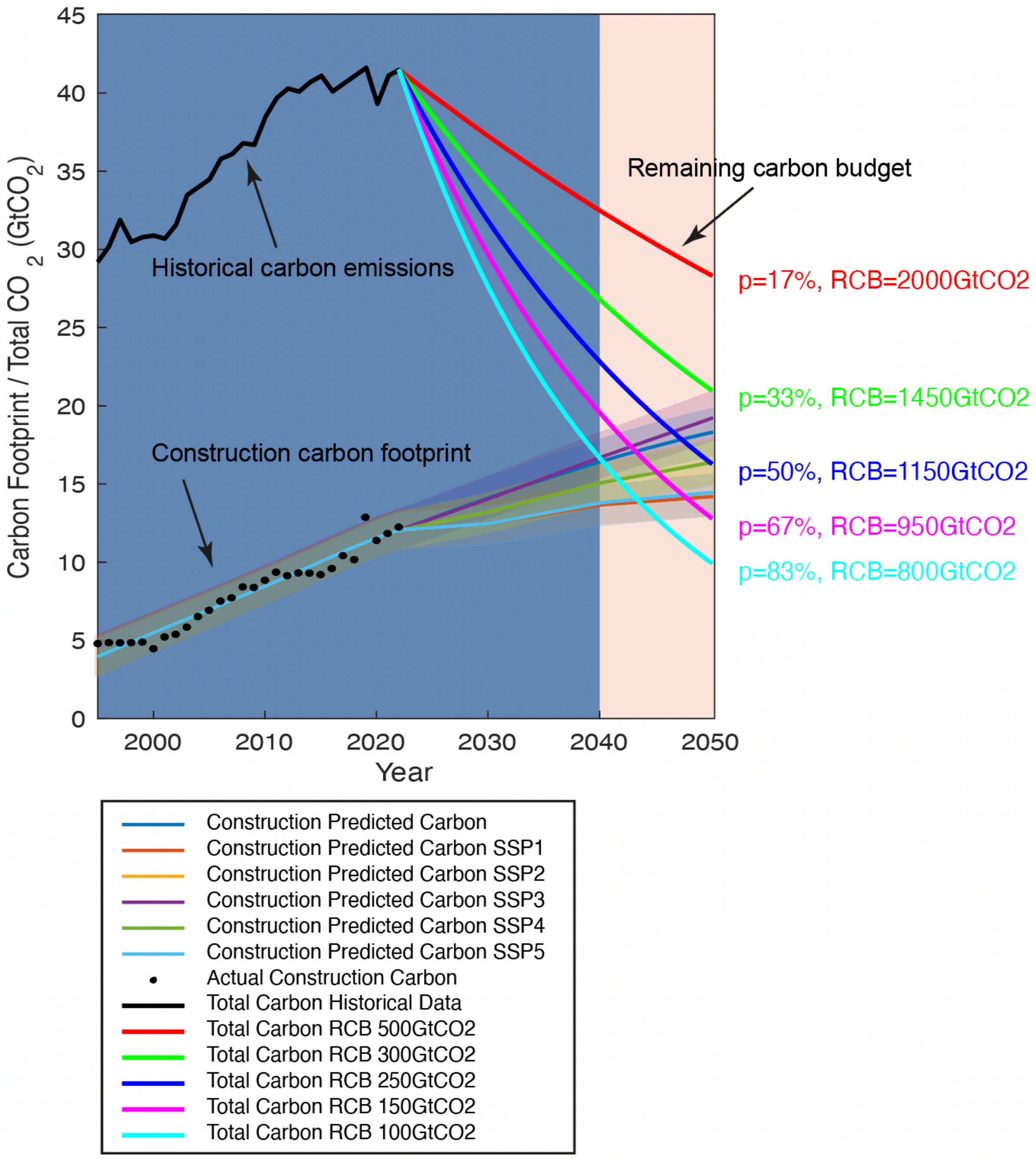

Fig. S2 Construction footprint projections and emission paths for reaching the 2 degrees Paris Agreement. Future annual carbon budget is modeled to achieve the 2 degrees Paris Agreement under 17%, 33%, 50%, 67%, and 83% possibilities. Future predictions of construction footprint is projected under different scenarios of Shared Socioeconomic Pathways (SSPs). Two pathways intersect in the starting from 2040. Beyond this, construction sector alone will use up all per-annum carbon budgets for 2 degrees.

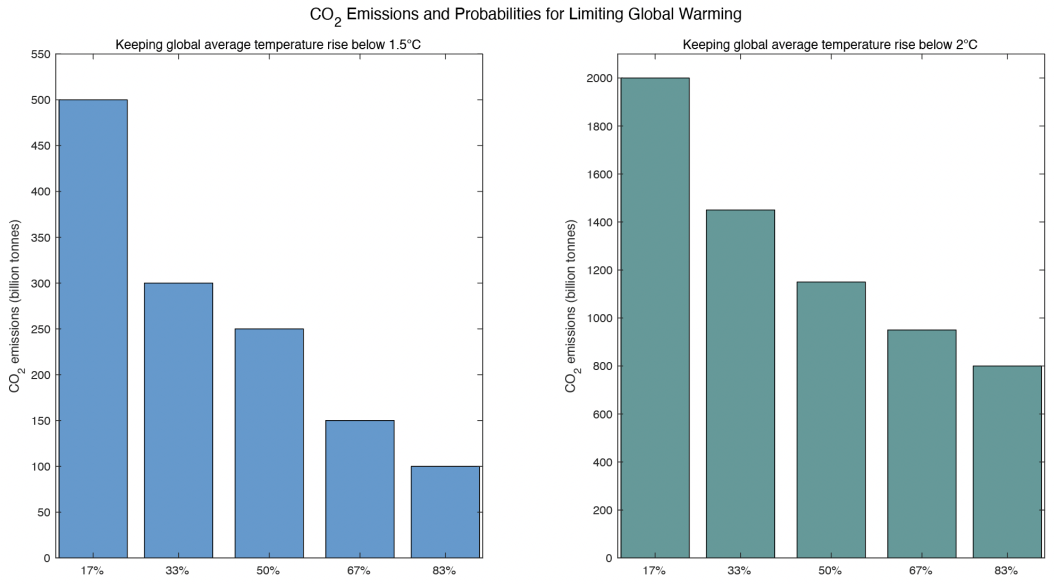

Fig. S3 Values for cumulative carbon budget for keeping within 1.5 and 2 degrees under various possibilities.

Supplementary References

1. Onat, N. C. & Kucukvar, M. Carbon footprint of construction industry: A global review and supply chain analysis. *Renew. Sustain. Energy Rev.* **124**, (2020).
2. Augiseau, V. & Barles, S. Studying construction materials flows and stock: A review. *Resour. Conserv. Recycl.* **123**, 153–164 (2017).
3. Tong, D. *et al.* Committed emissions from existing energy infrastructure jeopardize 1.5 °C climate target. *Nature* **572**, 373–377 (2019).
4. Davis, S. J., Caldeira, K. & Matthews, H. D. Future CO $_2$ Emissions and Climate Change from Existing Energy Infrastructure. *Science* **329**, 1330–1333 (2010).
5. Ma, M., Zhou, N., Feng, W. & Yan, J. Challenges and opportunities in the global net-zero building sector. *Cell Rep. Sustain.* **1**, 100154 (2024).
6. Mathur, V. S., Farouq, M. M. & Labaran, Y. H. The carbon footprint of construction industry: A review of direct and indirect emission. *J. Sustain. Constr. Mater. Technol.* **6**, 101–115 (2021).
7. Lanau, M. *et al.* Taking Stock of Built Environment Stock Studies: Progress and Prospects. *Environ. Sci. Technol.* **53**, 8499–8515 (2019).
8. Müller, D. B. *et al.* Carbon Emissions of Infrastructure Development. *Environ. Sci. Technol.* **47**, 11739–11746 (2013).
9. Huang, L., Krigsvoll, G., Johansen, F., Liu, Y. & Zhang, X. Carbon emission of global construction sector. *Renew. Sustain. Energy Rev.* **81**, 1906–1916 (2018).
10. Wu, X. *et al.* Extended carbon footprint and emission transfer of world regions: With both primary and intermediate inputs into account. *Sci. Total Environ.* **775**, (2021).
11. Ye, Q. *et al.* Allocating capital-associated CO2 emissions along the full lifespan of capital investments helps diffuse emission responsibility. *Nat. Commun.* **14**, 2727 (2023).
12. Zheng, X., Wang, R., Wood, R., Wang, C. & Hertwich, E. G. High sensitivity of metal footprint to national GDP in part explained by capital formation. *Nat. Geosci.* **11**, 269–273 (2018).
13. Wu, X. D., Guo, J. L., Li, C., Chen, G. Q. & Ji, X. Carbon emissions embodied in the global supply chain: Intermediate and final trade imbalances. *Sci. Total Environ.* **707**, 134670 (2020).
14. Fennell, P. S., Davis, S. J. & Mohammed, A. Decarbonizing cement production. *Joule* **5**, 1305–1311 (2021).
15. Ellis, L. D., Badel, A. F., Chiang, M. L., Park, R. J.-Y. & Chiang, Y.-M. Toward electrochemical synthesis of cement—An electrolyzer-based process for decarbonating CaCO3 while producing useful gas streams. *Proc. Natl. Acad. Sci.* **117**, 12584–12591 (2020).

16. Monteiro, P. J., Miller, S. A. & Horvath, A. Towards sustainable concrete. *Nat. Mater.* **16**, 698–699 (2017).

17. Stadler, K. *et al.* EXIOBASE 3: Developing a Time Series of Detailed Environmentally Extended Multi-Regional Input-Output Tables: EXIOBASE 3. *J. Ind. Ecol.* **22**, 502–515 (2018).

18. United Nations. *SEEA-W. System of Environmental-Economic Accounting for Water. United Nations Statistics Division.* (2012).

19. Kuenen, J., Usubiaga, A., Acosta-Fernández, J. & Merciai, S. SI_emissions Supporting Information for air emission accounts. *J. Ind. Ecol.* 29 (2018).

20. Eggleston, S., Buendia, L. & Miwa, K. *2006 IPCC Guidelines for National Greenhouse Gas Inventories*. (2006).

21. Merciai, S. & Schmidt, J. Methodology for the Construction of Global Multi-Regional Hybrid Supply and Use Tables for the EXIOBASE v3 Database: Methodology of MR-HSUTs for the EXIOBASE Database. *J. Ind. Ecol.* **22**, 516–531 (2018).

22. Figueres, C. *et al.* Three years to safeguard our climate. *Nature* **546**, 593–595 (2017).

23. Kikstra, J. S. *et al.* The IPCC Sixth Assessment Report WGIII climate assessment of mitigation pathways: from emissions to global temperatures. *Geosci. Model Dev.* **15**, 9075–9109 (2022).

24. Forster, P. M. *et al.* Indicators of Global Climate Change 2022: annual update of large-scale indicators of the state of the climate system and human influence. *Earth Syst. Sci. Data* **15**, 2295–2327 (2023).
