# Supplementary material for: Carbon footprint of the construction sector is projected to double by 2050 globally
Source: Commun Earth Environ. 2025 Oct 27;6(1):831. doi: 10.1038/s43247-025-02840-x (PMC12559003; doi:10.1038/s43247-025-02840-x)
Supplement: Supplementary file 2 — Description of Additional Supplementary Materials [file 43247_2025_2840_MOESM2_ESM.pdf]

Description of Additional Supplementary Files

File name: Supplementary Data 1

Description: tables on country, sector, and other input-output data details

File name: Supplementary Data 2

Description: tables on numerical outputs mentioned in the main text and supplementary information for this study

File name: Supplementary Data 3

Description: Tables on socio-economic datasets used in this study, regression results, and statistical tests.
